# Supplementary material for: Striatum supports fast learning but not memory recall
Source: Nature. 2025 May 7;643(8071):458–67. doi: 10.1038/s41586-025-08969-1 (PMC12244412; doi:10.1038/s41586-025-08969-1)
Supplement: Supplementary file 1 — Reporting Summary [file 41586_2025_8969_MOESM1_ESM.pdf]

nature portfolio

Corresponding author(s): Bernardo Sabatini

Last updated by author(s): Feb 13, 2025

Reporting Summary

Nature Portfolio wishes to improve the reproducibility of the work that we publish. This form provides structure for consistency and transparency in reporting. For further information on Nature Portfolio policies, see our Editorial Policies and the Editorial Policy Checklist.

Statistics

For all statistical analyses, confirm that the following items are present in the figure legend, table legend, main text, or Methods section.

n/a	Confirmed	
☐	☒	The exact sample size (*n*) for each experimental group/condition, given as a discrete number and unit of measurement
☐	☒	A statement on whether measurements were taken from distinct samples or whether the same sample was measured repeatedly
☐	☒	The statistical test(s) used AND whether they are one- or two-sided<br>*Only common tests should be described solely by name; describe more complex techniques in the Methods section.*
☐	☒	A description of all covariates tested
☐	☒	A description of any assumptions or corrections, such as tests of normality and adjustment for multiple comparisons
☐	☒	A full description of the statistical parameters including central tendency (e.g. means) or other basic estimates (e.g. regression coefficient) AND variation (e.g. standard deviation) or associated estimates of uncertainty (e.g. confidence intervals)
☐	☒	For null hypothesis testing, the test statistic (e.g. *F*, *t*, *r*) with confidence intervals, effect sizes, degrees of freedom and *P* value noted<br>*Give P values as exact values whenever suitable.*
☐	☒	For Bayesian analysis, information on the choice of priors and Markov chain Monte Carlo settings
☐	☒	For hierarchical and complex designs, identification of the appropriate level for tests and full reporting of outcomes
☐	☒	Estimates of effect sizes (e.g. Cohen's *d*, Pearson's *r*), indicating how they were calculated

Our web collection on statistics for biologists contains articles on many of the points above.

Software and code

Policy information about availability of computer code

Data collection	All custom code available at https://github.com/kimerein. Arduino code for behavior rig at https://github.com/kimerein/behaviorRig.<br>SpikeGLX (v3) and PlexControl (v1) for in vivo physiology. Igor (v6.02) for slice physiology. FlyCapture (v2) for high-speed video collection.<br>OlyVIA (v4.1) and VS120 or VS200 slide scanner for histology imaging.
Data analysis	Chronux (v3), DeepLabCut (v2.2), UltraMegaSort (v2000). All custom codes freely available online at https://github.com/kimerein. Tensor regression package at kimerein's Github and Richard Hakim's Github. Links here:<br>MATLAB analysis code: https://github.com/kimerein/integrate-phys-and-beh<br>https://github.com/kimerein/KR_Analysis_Toolbox<br>Python GLM code: https://github.com/kimerein/k-glm<br>Automated analysis of reaching in low-speed video:<br>https://github.com/kimerein/reach-behavior-analysis<br>https://github.com/kimerein/reachBehavior<br>Multi-unit processing of data from Plexon and WHISPER systems:<br>https://github.com/kimerein/MU-analysis<br>Python tensor regression: https://github.com/kimerein/tensor_regression<br>Photometry acquisition: https://github.com/kimerein/photometry<br>Arduino code: https://github.com/kimerein/behaviorRig<br>Python align high-speed video to events in behavior:<br>https://github.com/kimerein/integrate-phys-and-beh<br>UltraMegaSort spike sorting:<br>https://github.com/kimerein/Mat_Code/tree/master/UltraMegaSort

For manuscripts utilizing custom algorithms or software that are central to the research but not yet described in published literature, software must be made available to editors and reviewers. We strongly encourage code deposition in a community repository (e.g. GitHub). See the Nature Portfolio guidelines for submitting code & software for further information.

Data

Policy information about availability of data

All manuscripts must include a data availability statement. This statement should provide the following information, where applicable:

- Accession codes, unique identifiers, or web links for publicly available datasets
- A description of any restrictions on data availability
- For clinical datasets or third party data, please ensure that the statement adheres to our policy

We provide summary data sets at https://dataverse.harvard.edu/dataset.xhtml?persistentId=doi:10.7910/DVN/QPQEC9. We also provide example data sets for running the code at this same location. Because the total amount of raw data is well over 10 TB, and this volume is not well supported by the Harvard Dataverse, we have not uploaded all raw data to the Harvard Dataverse, but any raw data will be made freely available upon request.

Field-specific reporting

Please select the one below that is the best fit for your research. If you are not sure, read the appropriate sections before making your selection.

☒ Life sciences ☐ Behavioural & social sciences ☐ Ecological, evolutionary & environmental sciences

For a reference copy of the document with all sections, see nature.com/documents/nr-reporting-summary-flat.pdf

Life sciences study design

All studies must disclose on these points even when the disclosure is negative.

Sample size	Fig. 1: no pre-determination of sample size; choice of sample size: initially, we trained 6 mice in the behavior to ensure that animals could learn, but in the figure, we include the relevant mice collected after training mice for 4 years; sample size is sufficient, because clear differences in reaching across stages of learning. Fig. 2: no pre-determination of sample size; choice of sample size: we included all relevant mice after collecting data for 4 years; sample size is sufficient to see changes from first to second half of session, yet no observed change between control and interleaved pDMSt inhibition trials. Fig. 3: choice of sample size: we predetermined the sample size of mice in each cohort based on the max behavioral throughput given available experimenters; control and pDMSt silencing mice were run concurrently in batches of control plus pDMSt silencing mice; sample size is sufficient to see a learning deficit in pDMSt silencing mice (see Fig. 3 for statistics). Fig. 4: no pre-determination of sample size; choice of sample size: we included all relevant mice after collecting data for 4 years; sample size is sufficient, because bootstrap analysis indicates similar results and variability when we analyze the group of 37 mice or analyze the group of 11 mice, and hence adding more mice does not change the result. Fig. 5: no pre-determination of sample size; choice of sample size: after building a physiology rig, we recorded from all mice passing through the behavior pipeline over more than a year, before we switched to analysis of this data set without knowing the results; sample size is sufficient, because successful decoding of behavior condition indicates that sufficient data was acquired to decode held-out test data.
Data exclusions	Fig. 1: includes only mice that learned the task, excluding mice that failed to learn (but all mice are shown in final panel of Fig. 3), as planned. Fig. 2: all relevant mice and sessions included, as described in figure legend. Fig. 3: one mouse missing from recovery, as this mouse died. Fig. 4: all relevant mice included, except 5 mice were excluded from all analyses throughout the paper, because the behavior rig was not set up properly (as explained in Methods). Fig. 5: all relevant mice included.
Replication	Fig. 1: most mice learned the task, but others did not (Fig. 3 final panel shows all of the control mice that we attempted to train, including mice that failed to learn). Fig. 2: for in vivo physiology, we measured pDMSt silencing in two separate cohorts of mice recorded on different physiology rigs separated in time by more than one year -- hence, the pDMSt silencing method replicates; Fig. 2 suggests that the lack of effect of pDMSt silencing at the end of the session replicates across mice. Fig. 3: experimenters were blinded to genotype, and figure made with all relevant mice, but no specific replication of this result with another group of mice. Fig. 4: included all mice (n=37 mice), but no specific replication using another group of mice. Fig. 5: included all mice, and decoder trained on cross-validated data and tested on held-out data set.
Randomization	Genotype of the animal determined its belonging to the control or experimental (pDMSt silencing) group. We used mice as available including the double transgenic pDMSt silencing mice (Nkx2.1-Cre X ReaChR), preferring to use transgene-negative cage mates as the paired controls.
Blinding	Experimenters were blinded to animal genotype in Fig. 3. There was no blinding to genotype in the other figures, but note that training with the cue is automated and open-loop with little opportunity for experimenter influence.

Reporting for specific materials, systems and methods

We require information from authors about some types of materials, experimental systems and methods used in many studies. Here, indicate whether each material, system or method listed is relevant to your study. If you are not sure if a list item applies to your research, read the appropriate section before selecting a response.

Materials & experimental systems

n/a	Involved in the study
☐	☒ Antibodies
☒	☐ Eukaryotic cell lines
☒	☐ Palaeontology and archaeology
☐	☒ Animals and other organisms
☒	☐ Human research participants
☒	☐ Clinical data
☒	☐ Dual use research of concern

Methods

n/a	Involved in the study
☒	☐ ChIP-seq
☒	☐ Flow cytometry
☒	☐ MRI-based neuroimaging

Antibodies

Antibodies used

DARPP-32: Novus Biologicals primary antibody (Product # NB110-56929), GFP: anti-GFP from abcam (Product # ab13970), Anti-chicken: secondary antibody conjugated to Alexa488 from ThermoFisher (Product # A-11039), Anti-rabbit: secondary antibody conjugated to Alexa594 from ThermoFisher (Product # A-11012)

Validation

DARPP-32: There are no validation statements on the website, and we did not specifically validate the antibody. GFP: The abcam website states that the product was tested and does not cross-react with other proteins that differ from GFP by just a few point mutations (e.g., YFP). Anti-chicken: ThermoFisher website cites 2722 references. Anti-rabbit: ThermoFisher website cites 3293 references.

Animals and other organisms

Policy information about studies involving animals; ARRIVE guidelines recommended for reporting animal research

Laboratory animals

65 males and 62 females, including WT, Nkx2.1-Cre transgenic mouse line (Jackson Labs Stock #008661), Cre-On and Flp-On ReaChR transgenic mouse line (R26 LSL FSF ReaChR-mCitrine, Jackson Labs Stock #024846), Adora2a-Cre (GENSAT B6.FVB(Cg)-Tg(Adora2a-cre)KG139Gsat/Mmucd), D1-Cre (GENSAT B6.FVB(Cg)-Tg(Drd1a-cre)EY262Gsat/Mmcd). All mice (all strains) were initially injected between 2 and 6 months of age and trained 3 weeks after virus injection. Some animals continued to participate in the behavior up to 1.5 years of age.

Wild animals

no wild animals

Field-collected samples

no field-collected samples

Ethics oversight

President and Fellows of Harvard College IACUC

Note that full information on the approval of the study protocol must also be provided in the manuscript.
